# Avelumab in metastatic collecting duct carcinoma of the kidney: a case report

**DOI:** 10.1186/s13256-023-03973-3

**Published:** 2023-06-13

**Authors:** Nikolaos Pyrgidis, Ioannis Sokolakis, Gena Haltmair, Vitus Heller, Georgios Hatzichristodoulou

**Affiliations:** Department of Urology, ‘Martha-Maria’ Hospital Nuremberg, Stadenstr. 58, 90491 Nuremberg, Germany

**Keywords:** Avelumab, Immunotherapy, Collecting duct carcinoma of the kidney, Case report

## Abstract

**Background:**

Collecting duct carcinoma (CDC) of the kidney is associated with an aggressive course, limited response to treatment, and poor prognosis. Platinum-based chemotherapy is currently recommended as the first-line treatment in patients with metastatic CDC. Accumulating evidence supports the use of immunotherapy with checkpoint inhibitors as second-line therapy.

**Case presentation:**

In this case report, we describe the first case of avelumab administration due to disease progression during chemotherapy with gemcitabine and cisplatin in a Caucasian, 71-year-old man presenting with multiple metastases due to CDC of the kidney. The patient initially responded well to four cycles of chemotherapy and his performance status improved. After two additional chemotherapy cycles, the patient presented with new bone and liver metastases (mixed response to chemotherapy with an overall 6-month progression-free survival). We offered him avelumab as a second-line treatment in this setting. The patient received a total of three cycles of avelumab. The disease remained stable (no new metastases during treatment with avelumab), and the patient developed no complications. To alleviate his symptoms, radiation therapy for the bone metastases was decided. Despite successful radiation of the bone lesions and further improvement of symptoms, the patient developed hospital-acquired pneumonia and died approximately ten months after the initial diagnosis of CDC.

**Conclusions:**

Our findings suggest that the applied treatment modality with gemcitabine and cisplatin chemotherapy followed by avelumab was effective in terms of both progression-free survival and quality of life. Still, further studies assessing the use of avelumab in this setting are mandatory.

## Introduction

Collecting duct carcinoma (CDC) of the kidney, or Bellini tumor, is a rare subtype of renal cell carcinoma, accounting for less than two percent of all renal carcinomas. CDC is often diagnosed during the fifth decade of life and is commoner in men [1]. It is associated with a highly aggressive course, limited response to treatment, and poor prognosis. More than one-third of all patients with CDC is characterized with metastatic disease at diagnosis and have an overall survival of approximately 6 months [2]. CDC displays pathological, immunohistochemical, and cytogenetic features that are similar to urothelial carcinoma. Based on this observation, platinum-based chemotherapy is currently recommended as the first-line treatment in patients with metastatic CDC [3].

Accumulating evidence supports the use of immunotherapy with checkpoint inhibitors as second-line therapy in patients with metastatic CDC [4]. Individual case reports and retrospective case series have suggested that patients receiving substantial immunotherapy with antibodies that bind to the programmed cell death protein 1 (PD-1), programmed death-ligand 1 (PD-L1) or cytotoxic T-lymphocyte-associated protein 4 (CTLA-4) may benefit in terms of disease progression. Checkpoint inhibitors that have already been implemented in this setting include nivolumab, atezolizumab, and ipilimumab [5].

Avelumab has emerged as a promising antibody that binds to the PD-L1 that is currently approved for the management of metastatic urothelial carcinoma as maintenance therapy, as well as for disease progression during or after platinum-based chemotherapy [6]. Despite its efficacy in patients with metastatic urothelial carcinoma, studies administering avelumab in patients with metastatic CDC are currently lacking. In this scope, we report, to our knowledge, the first case of avelumab administration due to disease progression during chemotherapy with gemcitabine and cisplatin in a male patient presenting with metastatic CDC of the kidney.

## Case presentation

A 71-year-old man of Caucasian origin with no significant previous medical history and negative surgical history presented to his family doctor with progressive, unintentional weight reduction (Body mass index (BMI: 17.9 kg/m^2^)), fatigue, paraneoplastic fever, and dyspnea. In particular, the patients did not receive any medications regularly, his family history was negative, and he did not consume any alcohol or drugs. The patient was married with two children and had a good socioeconomic status. Other respiratory or renal symptoms were denied. These symptoms persisted for approximately 4 weeks. The patient’s ECOG performance status at baseline was three, he had normal vital signs (blood pressure: 131/84 mmHg, body temperature: 36.7 °C, pulse: 77 bpm, respiratory rate 17 breaths/minute with SpO_2_ of 96%), and had no abnormal findings in the laboratory studies (creatinine: 1.07 mg/dl, eGFR: 69 ml/min/1.73 m^2^, GOT: 17 U/l, GPT: 11 U/l, CRP: 4.7 mg/dl, TSH: 0.9 μU/ml, PSA: 2.5 ng/ml, Sodium: 139 mmol/l, Potassium: 4.4 mmol/l, WBC: 10.5/nl, Hemoglobin 12.6 g/dl and Thrombocytes: 360/nl).

Physical and ultrasound examination revealed a highly perfused left renal mass of approximately 8 × 3.5 cm. Computed tomography (CT) of the thorax and abdomen confirmed the presence of a suspicious left renal mass and revealed extensive regional lymphadenopathy, as well as a right adrenal mass and multiple lung lesions (cT2aN1M1). The patient was then referred to our institution for further management. CT-guided biopsies from the renal mass and a lung lesion were acquired, demonstrating the presence of primary metastatic CDC. After discussion of the case in a multidisciplinary tumor board, palliative chemotherapy with gemcitabine (1200 mg/m^2^) and cisplatin (70 mg/m^2^) based on the protocol for urothelial carcinoma was decided. Cytoreductive nephrectomy as individual concept was also suggested, but the patient rejected it.

**Fig. 1:**
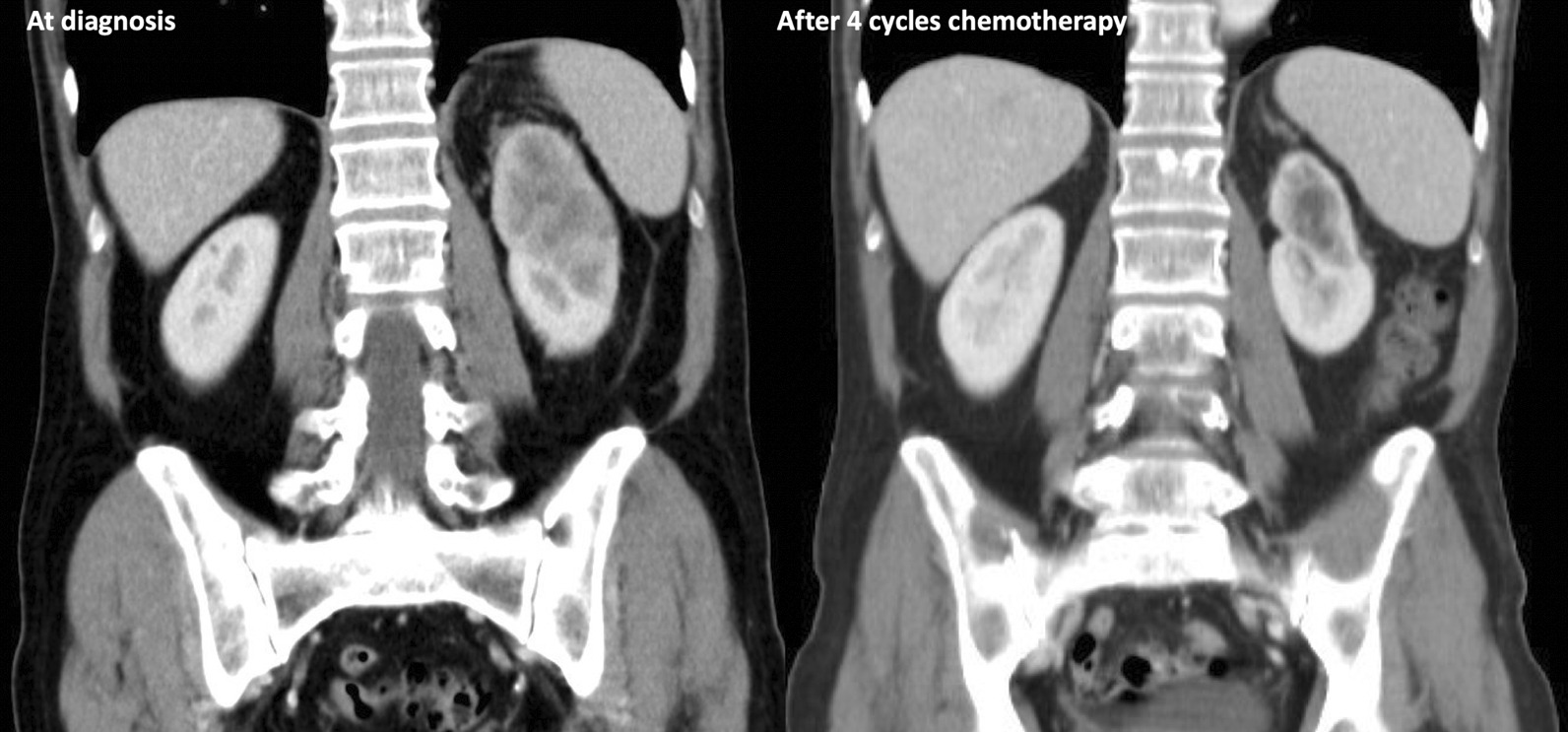
Pretreatment computed tomography scan with the presence of a large collecting duct carcinoma of the left kidney at diagnosis (left). Note the decrease in the size of the primary tumor after four cycles with gemcitabine/cisplatin chemotherapy (right)

**Fig. 2:**
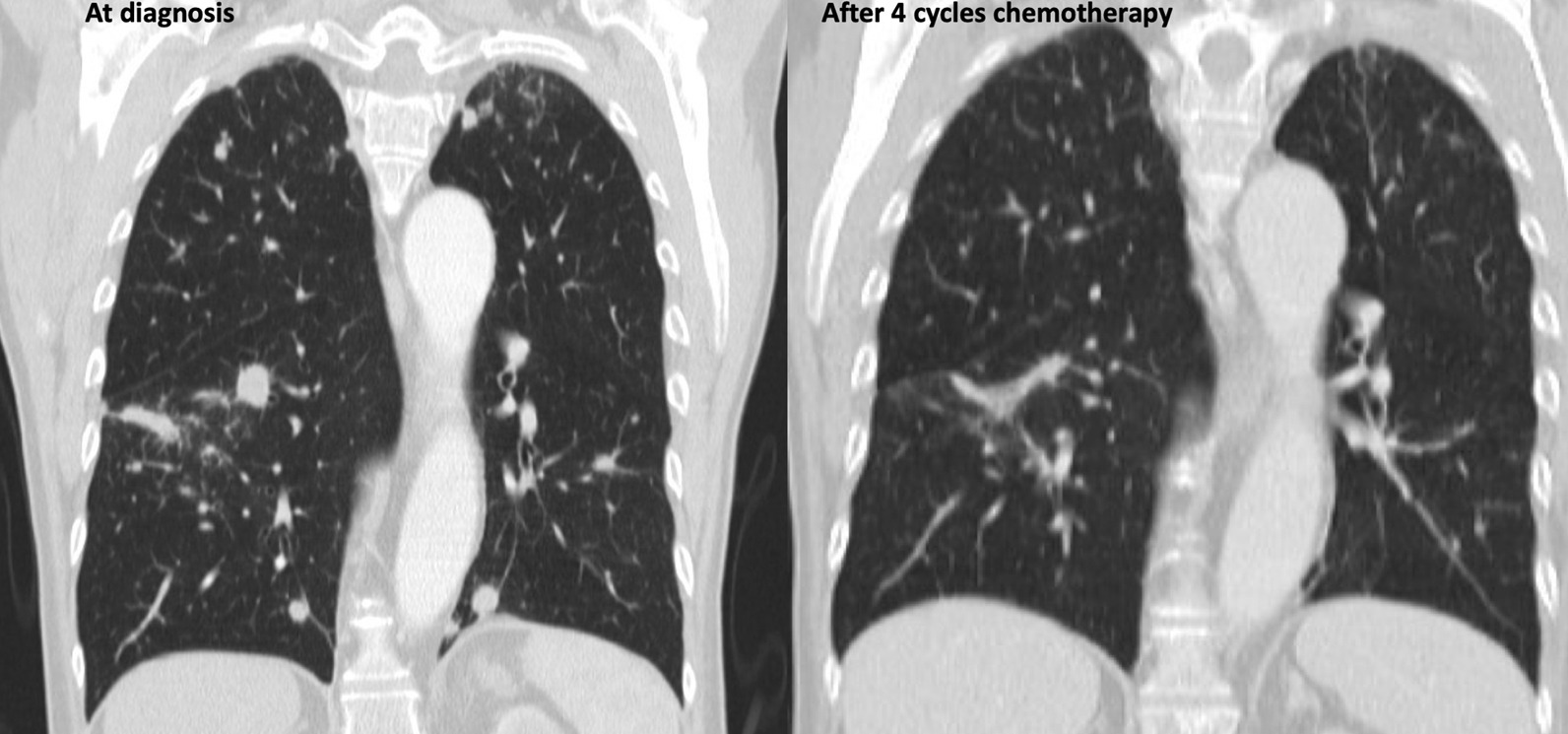
Pretreatment computed tomography scan with the presence of multiple lung metastases from the collecting duct carcinoma of the left kidney at diagnosis (left). Note the remission of the lung lesions after four cycles with gemcitabine/cisplatin chemotherapy (right)

**Fig. 3:**
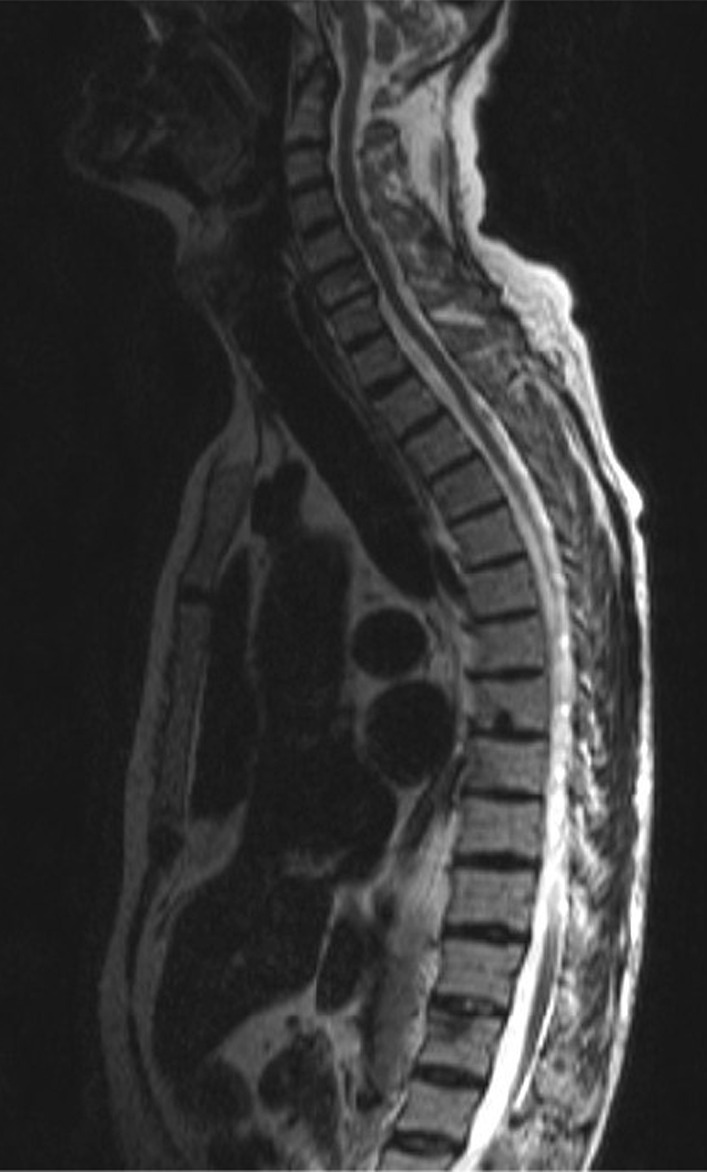
Magnetic resonance imaging of the thoracic spine with multiple bone metastases and no compression of the spinal cord

The patient showed an initial partial response to four cycles of chemotherapy and his ECOG performance status improved to one. Subsequently, a CT scan of the thorax and abdomen demonstrated an approximately 60% decrease in the size of the renal mass, an about 80% decrease of the lymphadenopathy, a complete remission of the right adrenal mass, and a complete remission of most lung lesions. No new metastases were detected (partial response to four cycles of chemotherapy). The effect of chemotherapy on the primary and metastatic lung lesions is illustrated in Figs. 1 and 2. Due to the satisfying response to treatment, two additional chemotherapy cycles with gemcitabine and cisplatin were performed. The patient continued to tolerate the chemotherapy well, but the final CT scan of the thorax and abdomen revealed the presence of multiple bone metastases in the thoracic and lumbar spine, as well as hepatic metastases (progression-free survival of 6 months). The other metastatic lesions displayed further regression (mixed response to the additional two cycles of chemotherapy) After a magnetic resonance imaging of the spine, compression of the spinal cord was excluded (Fig. 3) and the patient presented no sensory and motor deficits. At this point, the management options were discussed in detail with the patient to reach a shared decision-making. Due to mixed response of the metastases during platinum-based chemotherapy, we aimed for immunotherapy with a checkpoint inhibitor. Due to the promising good response rates of avelumab in metastatic urothelial carcinoma and its recent applications as maintenance therapy, we offered avelumab as a second line treatment to the patient [7].

The patient received a total of three cycles of avelumab (800 mg every 2 weeks). The disease remained stable (no new metastases during treatment with avelumab), and the patient developed no complications. Even though his performance status improved, he suffered from severe osteodynia due to bone metastases. In an attempt to alleviate his symptoms, radiation therapy for the bone lesions was decided and the patient was transferred to the radiation therapy clinic. Despite successful radiation of the bone lesions and further improvement of symptoms, the patient developed pneumonia with multidrug-resistant Enterococcus and Candida glabrata, which was fatal. The patient died approximately 10 months after the initial diagnosis of CDC.

## Discussion

The present case suggests that metastatic CDC requires a multidisciplinary approach due to its aggressivity involving urologists, oncologists, radiologists, and palliative caregivers. Even though our patient was already classified as a palliative case upon diagnosis and displayed worsening performance status, we immediately opted for chemotherapy which was beneficial in terms of both progression-free survival and quality of life. Still, given that the clinical outcomes for CDC are extremely poor, the patient displayed disease mixed response to chemotherapy. Recognizing that the available therapeutic options are restricted, we aimed for avelumab combined with radiation as second-line treatment. It should be highlighted that avelumab in this setting proved to be both safe and effective. Despite the good oncological response of avelumab, the patient died due to hospital-acquired pneumonia.

It should be noted that the first patient that received avelumab for CDC died from a cause not directly related to avelumab. Phase I to III studies on avelumab for urothelial carcinoma do not associate avelumab with increased risk for infections [8, 9]. No hospital-acquired infections or cases of pneumonia have been reported in these large-scale studies [10]. Additionally, a plethora of high-quality studies on avelumab used for other indications such as melanoma or lung carcinoma report similar findings [11]. Based on the previous notion, studies on other checkpoint inhibitors suggest that they should not be related to increased risk of infections [12]. Regarding pulmonary adverse events, checkpoint inhibitors have been associated with pneumonitis, which occurs in about 4% and seems to be similar across different PD-1 and PD-L1 inhibitors [13].

CDC is a rare and lethal renal tumor with limited response to pharmaceutical and surgical treatments. For patients with localized CDC, nephrectomy is the standard treatment. In the metastatic setting, conventional systemic treatments that are used for main types of renal cell carcinoma (like antiangiogenics or interferon) have shown limited efficacy against CDC [14]. Recently, a phase two trial evaluated cabozantinib as a first-line treatment for metastatic CDC, showing encouraging findings. More specifically, in 25 patients with metastatic CDC, the objective response rate after treatment with cabozantinib reached 35%, while the median progression-free and overall survival were 4 and 7 months, respectively [15]. Despite these promising findings, platinum-based chemotherapy remains, to date, the recommended first-line treatment modality in patients with metastatic CDC. Based on this recommendation, in our case, we opted for chemotherapy with gemcitabine and cisplatin, which offered a progression-free survival of 6 months.

In patients with metastatic CDC and disease progression during platinum-based chemotherapy, no recommendations exist, and the selection of treatment remains empirical. Avelumab may seem a promising checkpoint inhibitor in this setting. Avelumab is an IgG1 human antibody that binds to the PD-L1 and blocks the interaction between PD-L1 and PD-1. Physiologically, the binding of PD-L1 to PD-1 receptors in activated T-cells results in their inhibition, leading to immune suppression. Many tumors, including urothelial carcinoma, overexpress PD-L1 remaining unnoticed by the host’s immune system. Thus, by suppressing the binding of PD-L1 to PD-1 receptors, avelumab activates T-cells, resulting in the restoration of the host’s antitumor response [16]. Additionally, it seems that avelumab further stimulates the host's antitumor response by inhibiting the interaction of PD-L1 with B7.1 receptors, which are expressed on antigen-presenting cells and T-cells. Importantly, avelumab can engage the innate immune system by inducing antibody-dependent cell-mediated cytotoxicity through natural killer cells, resulting in tumor cell lysis without the need for T-cell adaptive immunity involvement [17]. The latter is a unique characteristic of avelumab compared to other checkpoint inhibitors targeting PD-1 or PD-L1 receptors. Other checkpoint inhibitors do not trigger antibody-dependent cell-mediated cytotoxicity either because they belong to the IgG4 subclass (like nivolumab or pembrolizumab) or because they are not completely human antibodies (like atezolizumab or durvalumab). Therefore, avelumab is the only PD-1/PD-L1 inhibitor that induces the host's antitumor response via two different mechanisms, potentially leading to an enhanced clinical efficacy [18].

It should be highlighted that it remains unclear whether systematic therapies approved for the main types of renal cell carcinoma or urothelial carcinoma are indeed effective in patients with CDC. Thus, it is mandatory to design and implement studies focusing on the genomic insights of the CDC in an attempt to reveal novel therapeutic targets for this rare form of renal cell carcinoma [19]. Nevertheless, the extremely low incidence of CDC makes it difficult to conduct high-quality studies and, thus, multi-institutional data are necessary. Considering that avelumab may be a promising checkpoint inhibitor, multi-institutional phase two studies evaluating avelumab as a first- or second-line treatment for metastatic CDC are needed. Overall, it should be stressed that we are currently experiencing rapid growth in research on checkpoint inhibitors, which could offer the potential to augment patient care. Therefore, studies on checkpoint inhibitors may provide further insights into the management of CDC and other renal tumors.

## Conclusion

CDC is a highly aggressive renal tumor with an extremely poor prognosis. In this report, we present a palliative case of CDC with multiple metastases that responded well to chemotherapy with gemcitabine and cisplatin, and subsequently received immunotherapy with avelumab. Considering the extensive metastatic disease and the performance status of the patient upon diagnosis, the applied treatment modality was effective in terms of both survival and quality of life. Still, it should be noted that all available treatment modalities offer a very short duration of response. This observation reinforces the idea that personalized medicine is necessary for the management of rare and aggressive tumors. Therefore, both preclinical studies focusing on the pathological, immunohistochemical, and cytogenetic features of CDC and clinical studies assessing novel treatment modalities are mandatory in an attempt to expand our therapeutic armamentarium in patients with CDC.

## Data Availability

The data that support the findings of this study are available from the corresponding author, Nikolaos Pyrgidis, upon reasonable request.
